# What is the impact of chronic kidney disease stage and cardiovascular disease on the annual cost of hospital care in moderate-to-severe kidney disease?

**DOI:** 10.1186/s12882-015-0054-0

**Published:** 2015-04-29

**Authors:** Seamus Kent, Iryna Schlackow, Jingky Lozano-Kühne, Christina Reith, Jonathan Emberson, Richard Haynes, Alastair Gray, Alan Cass, Colin Baigent, Martin J Landray, William Herrington, Borislava Mihaylova, on behalf of the SHARP Collaborative Group

**Affiliations:** Health Economics Research Centre (HERC), Nuffield Department of Population Health, University of Oxford, Oxford, UK; Clinical Trial Service Unit and Epidemiological Studies Unit (CTSU), Nuffield Department of Population Health, University of Oxford, Oxford, UK; Menzies School of Health Research, Sydney, Australia

**Keywords:** Cardiovascular event, Chronic kidney disease, Annual hospital costs, Renal replacement therapy, Resource use

## Abstract

**Background:**

Reliable estimates of the impacts of chronic kidney disease (CKD) stage, with and without cardiovascular disease, on hospital costs are needed to inform health policy.

**Methods:**

The Study of Heart and Renal Protection (SHARP) randomized trial prospectively collected information on kidney disease progression, serious adverse events and hospital care use in a cohort of patients with moderate-to-severe CKD. In a secondary analysis of SHARP data, the impact of participants’ CKD stage, non-fatal cardiovascular events and deaths on annual hospital costs (i.e. all hospital admissions, routine dialysis treatments and recorded outpatient/day-case attendances in United Kingdom 2011 prices) were estimated using linear regression.

**Results:**

7,246 SHARP patients (2,498 on dialysis at baseline) from Europe, North America, and Australasia contributed 28,261 years of data. CKD patients without diabetes or vascular disease incurred annual hospital care costs ranging from £403 (95% confidence interval: 345-462) in CKD stages 1-3B to £525 (449-602) in CKD stage 5 (not on dialysis). Patients in receipt of maintenance dialysis incurred annual hospital costs of £18,986 (18,620-19,352) in the year of initiation and £23,326 (23,231-23,421) annually thereafter. Patients with a functioning kidney transplant incurred £24,602 (24,027-25,178) in hospital care costs in the year of transplantation and £1,148 (978-1,318) annually thereafter. Non-fatal major vascular events increased annual costs in the year of the event by £6,133 (5,608-6,658) for patients on dialysis and by £4,350 (3,819-4,880) for patients not on dialysis, and were associated with increased costs, though to a lesser extent, in subsequent years.

**Conclusions:**

Renal replacement therapy and major vascular events are the main contributors to the high hospital care costs in moderate-to-severe CKD. These estimates of hospital costs can be used to inform health policy in moderate-to-severe CKD.

**Electronic supplementary material:**

The online version of this article (doi:10.1186/s12882-015-0054-0) contains supplementary material, which is available to authorized users.

## Background

In the United Kingdom (UK), the age-standardized prevalence of chronic kidney disease (CKD) stages 3 to 5 not on renal replacement therapy (RRT) has been estimated at 11% for females and 6% for males [1]. The prevalence of end-stage renal disease requiring RRT is 0.1%, of which 49% have a functioning kidney transplant, 44% are on hemodialysis and 8% use peritoneal dialysis [2]. The annual cost of CKD to the National Health Service (NHS) in England in 2009-2010 was estimated at £1.45 billion (1.3% of total NHS expenditure), with the cost of RRT accounting for more than half of this total [3]. The increased risk of vascular and nonvascular morbidity and mortality conferred by CKD [4-8] is a key determinant of these substantial healthcare costs.

The estimate of NHS expenditure on CKD in England in Kerr et al. [3] was derived using a variety of secondary sources, including the primary care Quality and Outcomes Framework, NHS Blood and Transplant, the UK Renal Registry, and NHS Reference costs. The costs of cardiovascular disease attributable to CKD were calculated by attaching unit costs estimated in general population settings to the estimated excess numbers of myocardial infarctions and strokes in people with CKD. A more reliable method is to use individual CKD patient data to directly estimate per person costs of the disease. An individual patient data approach has been employed in a number of CKD cost studies [9-14]; however, none of these estimated the separate contributions of CKD progression and cardiovascular events on hospital care costs. We use individual patient data from the Study of Heart and Renal Protection (SHARP) to estimate the annual hospital care costs (defined as the cost of all hospital admissions, routine dialysis treatments and any recorded outpatient/day-case attendances) associated with CKD stage and cardiovascular complications in CKD, and to develop a UK annual cost prediction model in CKD.

## Methods

### SHARP study population

Details of the design of the SHARP trial and its main results have been reported previously [15,16]. Briefly, individuals aged 40 years or over were eligible to participate if they had more than one previous measurement of serum or plasma creatinine of at least 1.7 mg/dL (150 μmol/L) in men or 1.5 mg/dL (130 μmol/L) in women, or were receiving maintenance dialysis. Patients with prior myocardial infarction or coronary revascularization were excluded. Between 2003 and 2006, 9,270 patients from 18 countries in Europe, North America, Asia, and Australasia provided written informed consent and were randomized and followed for a median of 4.9 years until the end of the study in 2010. Ethical approval for SHARP was given by the Thames Valley Multicentre Research Ethics committee. The 1,928 (21%) patients recruited in Asia were excluded from the analysis presented here because there were important differences in the case-mix of hospital events and substantially lower rates of hospitalization for major vascular events, compared to patients from other regions.

### Serious adverse events

Following randomization, patients were followed up at study clinics at 2 and 6 months, and then every 6 months. At each follow-up visit, information on all hospital admissions and possible study outcomes was recorded. If a patient became unwilling or unable to attend follow-up visits, information was sought from them (or a relative or carer) by telephone or from their doctor until the scheduled study end. Further information was sought from hospital records and other appropriate sources for all events that might represent an important study outcome. Trained clinicians adjudicated important study outcomes in accordance with pre-specified definitions. All diagnoses were subsequently coded into the International Classification of Diseases (ICD-10) system and all procedures into the Operating Code Supplement (OPCS-4.5) system. For the purpose of the hospital cost analysis, follow-up time was divided into annual periods from the date of randomization to the end of study. Only fully observed annual periods and annual periods shorter than one year due to death were included in the cost analysis.

### Cardiovascular disease

Cardiovascular disease and vital status were determined for each patient at the end of each annual period and categorized into a mutually exclusive hierarchy (in descending order): i) vascular death during the current annual period; ii) non-vascular death during the current annual period; iii) a non-fatal major vascular event (MVE: defined as coronary death, non-fatal myocardial infarction, any arterial revascularization procedure, or stroke) during the current annual period; iv) most recent non-fatal MVE in the preceding annual period; v) most recent non-fatal MVE occurred in the annual period before last; vi) most recent non-fatal MVE occurred more than two annual periods previously; vii) prior vascular disease at randomization but no MVE since; and, viii) no vascular disease at randomization and no subsequent MVE.

### Chronic kidney disease staging

Renal status was determined at each follow-up visit using local measurements of serum/plasma creatinine, or, where relevant, current RRT modality. For each annual period, the mean of all recorded creatinine results in the period was used to calculate the CKD Epidemiology Collaboration (CKD-EPI) estimated glomerular filtration rate (eGFR) [17] and categorize patients into the following mutually exclusive ‘stages’: CKD stage 1-3B (eGFR ≥30 ml/min/1.73 m^2^); CKD stage 4 (eGFR ≥15, <30 ml/min/1.73 m^2^); CKD stage 5 not on RRT (eGFR <15 ml/min/1.73 m^2^); maintenance dialysis initiated in the current annual period; maintenance dialysis initiated in a preceding annual period; kidney transplantation in the current annual period; or, functional kidney transplant from an earlier annual period. In the cases in which information was absent, the latest information on CKD stage was carried forward for up to two years (for 3% of annual periods).

### Hospital care use and costs

All serious adverse events related to hospital care use were classified into one of four mutually exclusive categories: i) atherosclerotic events; ii) non-atherosclerotic vascular events; iii) renal events; or iv) other events (i.e. non-vascular, non-renal events) [see Additional file 1: Table S1 for full description]. Hospital episodes (inpatient admissions, day case, or outpatient attendances) were formed from serious adverse events with overlapping durations. Where a hospital episode involved more than one event, the episode was classified according to the (descending) hierarchical order above. Each patient’s hospital episodes, alongside information on their age and comorbidities, were mapped into 2010-11 UK Healthcare Resource Groups [18] with UK Hospital Trust costs [19] (the approach to costing recommended by the National Institute for Health and Care Excellence [20]). Although the data collected in the study did not allow the identification of finished consultant episodes (FCE) for hospital care, which is used as the unit for costing healthcare activity in the UK, it should be noted that more than 90% of hospital admissions in the UK contain only one FCE [21]. We also established the cost of the episode using the most resource intensive of the events experienced, further reducing the extent of any underestimation.

Information on routine dialysis sessions was not recorded in SHARP. Instead, the 2010-11 UK costs of maintenance dialysis treatment were estimated for each patient based on their six-monthly dialysis status assuming thrice weekly hemodialysis sessions (£24,973 per annum) and daily peritoneal dialysis sessions (£20,449 per annum) [19,22].

### Statistical methods

A wide range of baseline socio-demographic and clinical patient characteristics, annually updated CKD stage and annually updated cardiovascular event history were related to annual hospital care costs using a linear regression model. The final statistical model was selected from a wider range of candidate models using common specification tests [23] and a comparative assessment of each model’s predictive performance. A backward-stepwise selection procedure using statistical criteria was used to retain important covariates and interactions (see Additional file 1: Statistical appendix for full details). Standard errors of the parameter estimates in the regression model were adjusted for the multiple annual cost observations per patient [24]. Mean annual hospital costs were calculated for patient profiles by CKD stage and history of cardiovascular events. Mean absolute prediction error was calculated using cross-validation based on 1,000 randomly split samples [25]. All analyses were performed using R 3.0.0 [26].

## Results

After excluding the 5,847 annual periods with incomplete information due to the end of study follow up, 28,261 annual periods of observation were available across 7,246 patients recruited in non-Asian countries. Of these patients, 5,083 (70%) were recruited in Europe, 1,302 (18%) in Australasia and 861 (12%) in North America. Mean age was 63 (standard deviation 12) years, 4,652 (64%) were male, 1,116 (15%) had established vascular disease and 1,439 (20%) had diabetes mellitus at baseline. CKD stage data were recorded in 27,327 (97%) annual periods. 4,739 (65%) patients were not on RRT, of which 1,494 (32%) were in CKD stages 1-3B (mean eGFR = 37.6 mL/min/1.73 m^2^), 2,228 (47%) in CKD stage 4 and 1,017 (21%) in CKD stage 5. 2,498 (34%) patients were on maintenance dialysis at baseline, of which 2,076 (83%) were on hemodialysis and 422 (17%) on peritoneal dialysis (Table 1). By the end of the cost study period, 4,013 (55%) patients were receiving RRT, including 800 (79%) of those with CKD stage 5 at baseline, 621 (28%) of those with CKD stage 4 and 85 (6%) of those in CKD stages 1-3B (Additional file 1: Table S2). During the study follow-up there were 994 (3.5% per year) kidney transplants and 1,362 (4.8% per year) patients started (or restarted following a transplant failure) maintenance dialysis (Table 2).

**Table 1:** **Baseline characteristics of SHARP patients included in the cost analysis**

	**By baseline CKD stage** ^**1**^				**Overall**
	**CKD 1-3B (eGFR: ≥30)** ^**2**^	**CKD 4 (eGFR: ≥15, <30)**	**CKD 5 (eGFR: <15 not on dialysis)**	**On dialysis**	
Number of patients	1,494	2,228	1,017	2,498	7,246
Men	1,078 (72%)	1,368 (61%)	599 (59%)	1,600 (64%)	4,652 (64%)
Age (years)	62 (11)	65 (12)	63 (12)	61 (12)	63 (12)
Prior vascular disease	189 (13%)	350 (16%)	147 (14%)	428 (17%)	1,116 (15%)
Prior diabetes mellitus	268 (18%)	454 (20%)	169 (17%)	546 (22%)	1,439 (20%)
Current smoker	205 (14%)	276 (12%)	133 (13%)	432 (17%)	1,048 (14%)
Body-mass index (kg/m^2^)	28.4 (5.3)	28.2 (5.8)	27.5 (5.5)	27.0 (5.9)	27.8 (5.7)
Diastolic blood pressure (mm Hg)	80 (13)	79 (13)	80 (12)	77 (13)	79 (13)
Systolic blood pressure (mm Hg)	138 (20)	138 (20)	141 (20)	137 (24)	138 (21)
Total cholesterol (mmol/L)	5.1 (1.1)	5.1 (1.1)	4.9 (1.2)	4.6 (1.2)	4.9 (1.2)
LDL cholesterol (mmol/L)	3.0 (0.8)	2.9 (0.8)	2.8 (0.9)	2.6 (0.9)	2.8 (0.9)
HDL cholesterol (mmol/L)	1.2 (0.3)	1.2 (0.3)	1.1 (0.3)	1.1 (0.4)	1.1 (0.3)
Triglycerides (mmol/L)	2.3 (1.6)	2.3 (1.4)	2.2 (1.4)	2.3 (1.9)	2.3 (1.6)
Renal status					
On dialysis	-	-	-	2,498 (100%)	2,498 (34%)
Hemodialysis	-	-	-	2,076 (83%)	2,076 (29%)
Peritoneal dialysis	-	-	-	422 (17%)	422 (6%)
Not on dialysis	1,494 (100%)	2,228 (100%)	1,017 (100%)	-	4,739 (65%)
Estimated glomerular filtration rate (mL/min/1.73^2^)	37.6 (6.5)	22.5 (4.3)	10.8 (2.7)	-	24.8 (10.9)
Urinary albumin:creatinine ratio (mg/g)^3^					
Median (IQR)	75 (16–328)	155 (36–592)	397 (124–1,204)	-	156 (35–626)
<30	459 (36%)	426 (22%)	62 (7%)	-	947 (23%)
≥30, ≤ 300	496 (38%)	820 (42%)	332 (37%)	-	1,648 (40%)
>300	336 (26%)	722 (37%)	511 (56%)	-	1,569 (38%)
Not available	203	260	112	-	584
Region of recruitment					
Europe	1,022 (68%)	1,546 (69%)	730 (72%)	1,779 (71%)	5,083 (70%)
Australasia	260 (17%)	446 (20%)	187 (18%)	408 (16%)	1,302 (18%)
North America	212 (14%)	236 (11%)	100 (10%)	311 (12%)	861 (12%)

Data are n (%), mean (SD) or median (IQR); CKD = chronic kidney disease.

^1^9 patients who received a transplant prior to randomization were excluded from the tabulation by baseline CKD stage.

^2^Predominantly CKD stage 3B (eGFR ≥30 to <45 ml/min/1.73m^2^).

^3^Percentages exclude patients for whom data were not available.

**Table 2:** **Number of patient years with events and use of renal replacement therapy by baseline CKD stage**

	**Baseline CKD stage** ^**1**^				**Overall**
	**CKD 1-3B** ^**2**^	**CKD 4**	**CKD 5 not on dialysis**	**On dialysis**	
Number of patients	1,494	2,228	1,017	2,498	7,246
Vascular death	36 (0.6%)	92 (1.0%)	86 (2.2%)	235 (2.5%)	449 (1.6%)
Non-vascular death	97 (1.6%)	219 (2.5%)	151 (3.8%)	453 (4.9%)	920 (3.3%)
Non-fatal major vascular event in the current annual period	93 (1.5%)	222 (2.5%)	128 (3.2%)	385 (4.1%)	828 (2.9%)
Kidney transplantation in the current annual period	14 (0.2%)	142 (1.6%)	213 (5.4%)	625 (6.7%)	994 (3.5%)
On functioning kidney transplant from an earlier annual period	9 (0.1%)	126 (1.4%)	335 (8.5%)	1,170 (12.5%)	1,663 (5.9%)
Dialysis initiated during the current annual period	77 (1.3%)	548 (6.2%)	718 (18.2%)	18 (0.2%)	1,362 (4.8%)
On dialysis since an earlier annual period	44 (0.7%)	609 (6.9%)	1,337 (33.8%)	7,526 (80.6%)	9,516 (33.7%)

Data are the number of annual periods in which the defined event occurs or the RRT status, and percentage of all annual periods. In each annual period, each participant on renal replacement therapy is classified into one of: (1) kidney transplantation in current annual period; (2) functioning kidney transplantation from an earlier annual period; (3) dialysis initiated during the current annual period; and (4) on dialysis since an earlier annual period.

CKD = chronic kidney disease.

^1^9 patients who received a transplant prior to randomization were excluded from the tabulation by baseline CKD stage.

^2^Predominantly CKD stage 3B (eGFR ≥30 to <45 ml/min/1.73m^2^).

A total of 21,157 hospital care episodes were recorded across the 7,246 study patients, an average of 0.77 episodes per patient per year (excluding routine dialysis sessions). These included 0.33 episodes per year for non-vascular/non-renal events, 0.32 episodes per year for renal events, and 0.06 episodes per year for each of atherosclerotic and non-atherosclerotic vascular events per patient. Worse kidney function at baseline was associated with increased rates of hospital episodes of all types. Non-vascular/non-renal episodes per patient-year ranged from 0.24 among those in CKD stages 1-3B to 0.45 in those on dialysis. Renal episodes (excluding routine dialysis) were lowest in those in CKD stages 1-3B (0.06 per patient per year [ppy]) and greatest in those with CKD stage 5 not on RRT (0.59 ppy). Hospital episodes for both atherosclerotic and non-atherosclerotic vascular disease per patient-year more than doubled from 0.02-0.03 ppy among those in CKD stages 1-3B to 0.08 ppy in those on dialysis at baseline (Additional file 1: Table S3).

Hospital care costs were incurred in 16,227 (57%) annual periods, ranging from 24% of annual periods among those in CKD stages 1-3B at baseline to 91% of annual periods in those on maintenance dialysis at baseline (Table 3). The average observed annual cost of hospital care (including routine dialysis costs) was £9,977 (SE 69), ranging from £1,055 (SE 46) for those in CKD stages 1-3B through £12,952 (SE 185) for those with CKD stage 5 not on RRT, to £20,511 (SE 93) for those on maintenance dialysis at baseline.

**Table 3:** **Observed annual hospital costs by baseline CKD stage**

**Baseline CKD stage** ^**1**^	**Number of patients**	**Years of follow-up**	**Years of follow-up with hospital use, n (%)**	**Mean (SE) hospital cost per person-year of follow-up**
CKD 1-3B^2^	1,494	6,077	1,447 (24%)	£1,055 (46)
CKD 4	2,228	8,867	3,379 (38%)	£3,694 (84)
CKD 5 not on dialysis	1,017	3,954	2,849 (72%)	£12,952 (185)
Dialysis	2,498	9,339	8,543 (91%)	£20,511 (93)
**All patients**	**7,246**	**28,261**	**16,227 (57%)**	**£9,977 (69)**

CKD = chronic kidney disease; SE = standard error.

^1^9 patients who received a transplant prior to randomization were excluded from the tabulation by baseline CKD stage.

^2^Predominantly CKD stage 3B (eGFR ≥30 to <45 ml/min/1.73m^2^).

From the large number of characteristics initially considered for inclusion in the cost model, only annually updated CKD stage; history of cardiovascular disease and prior diabetes mellitus at baseline; and interactions between receiving dialysis and death and receiving dialysis and experiencing a non-fatal major vascular event in the current annual period, were retained in the model. After accounting for these characteristics, patients with CKD but without diabetes or vascular disease were estimated to incur annual hospital care costs ranging from £403 (95% confidence interval 345-462) in CKD stages 1-3B to £525 (449-602) in CKD stage 5. This was mainly due to 19% of annual periods in CKD stage 5 containing admissions for the formation/insertion of dialysis access, compared to 2% in CKD stage 4. CKD patients in receipt of maintenance dialysis incurred £18,986 (18,620-19,352) costs in the year of initiation and £23,326 (23,231-23,421) in each subsequent year. Average annual hospital care costs in the year of transplantation were £24,602 (24,027-25,178), largely due to the costs of admission for the transplant operation itself (UK hospital admission cost for a kidney transplant was £20,798 [for a live donor] or £19,417 [for a deceased donor; excluding all costs of donation and drugs] [19]), and £1,148 (978-1,318) in subsequent years (Table 4).

**Table 4:** **Estimated annual UK hospital care costs of chronic kidney disease patients by stage of chronic kidney disease and presence of cardiovascular complications**

**Annual hospital care costs (£, 95% CI) in the absence of diabetes and cardiovascular complications**	
In CKD stage 1-3B^1^	£403 (345–462)
In CKD stage 4	£393 (343–444)
In CKD stage 5	£525 (449–602)
On functioning kidney transplant from the current annual period	£24,602 (24,027-25,178)
On functioning kidney transplant from an earlier annual period	£1,148 (978–1,318)
On maintenance dialysis initiated in the current annual period^2^	£18,986 (18,620-19,352)
On maintenance dialysis initiated in an earlier annual period^2^	£23,326 (23,231-23,421)
**Additional annual hospital care costs associated with diabetes, cardiovascular complications and death (£, 95% CI)**	
With prior diabetes^3^	£171 (54–288)
Died from vascular cause in the current annual period	£1,137 (469–1,804)
Died from non-vascular cause in the current annual period	£1,391 (1,020-1,763)
Experienced non-fatal MVE during the current annual period, not on maintenance dialysis	£4,350 (3,819-4,880)
Experienced non-fatal MVE during the current annual period; on maintenance dialysis	£6,133 (5,608-6,658)
Latest non-fatal MVE experienced in the preceding annual period	£738 (351–1,126)
Other vascular disease (defined as with vascular disease at baseline or experienced latest non-fatal MVE two or more years previously)	£172 (57–286)

CKD = chronic kidney disease; MVE = major vascular events; CI = confidence interval.

^1^Predominantly CKD stage 3B (eGFR ≥30 to <45 ml/min/1.73m^2^).

^2^Annual costs associated with maintenance dialysis are reduced by £8,802 (8,172-9,432) in years of death.

^3^Defined at baseline.

Death from vascular and nonvascular causes increased hospital costs in the year of death by £1,137 (469-1,804) and £1,391 (1,020-1,763) respectively. Non-fatal MVEs increased annual hospital care costs in the year of the event by £4,350 (3,819-4,880) for individuals not on dialysis (including those with a functioning transplant) and by £6,133 (5,608-6,658) for those receiving dialysis. The effect of a vascular event on hospital costs remained, but was diminished, in subsequent years: in the absence of further MVEs, the effect of a non-fatal MVE on hospital costs in the following year was £738 (351-1,126) and £172 (57-286) in each year thereafter. Having prior diabetes at entry into the study was associated with additional annual hospital costs of £171 (54-288) (Table 4). Estimates of annual hospital costs were similar in a sensitivity analysis limited to data from the 1,910 patients recruited in the United Kingdom. The inclusion of albuminuria into the cost model was not found to offer further predictive information for determining hospital care costs. The final model had good aggregate and individual level predictive accuracy, with the mean absolute prediction error estimated at 20% of the mean cost (see Additional file 1: Table S5).

## Discussion

A regression analysis on individual data from more than 7,000 CKD patients was used to estimate the annual hospital care costs associated with CKD stage and cardiovascular disease. The reported hospital cost model (Table 4) allows the annual hospital care costs (in UK 2011 prices) to be estimated for a patient with moderate-to-severe CKD if the CKD stage, cardiovascular disease history, diabetes mellitus and vital status are known (see Additional file 1: Table S4 for examples).

The predicted annual costs capture not only the direct cost of an event (e.g. myocardial infarction or stroke), but also costs of other related health problems in the year. Consequently, the average estimated additional cost of a major vascular event in the annual period in which it occurred (£6,133 for those on dialysis and £4,350 for those not) are substantially higher than the separate reference costs of vascular events (e.g. the cost for a hospital admission for myocardial infarction in 2011 was £1,410 and for a stroke in patients on and not on dialysis was £2,567 and £1,773 respectively [19]). Moreover, experiencing a major vascular event was found to have an impact on hospital care costs in subsequent years, even in the absence of further such events.

Renal replacement therapy has a substantially greater impact on annual hospital costs than vascular events. Although the annual hospital care cost in the year of kidney transplantation was about 10% greater than the cost in a year of initiating dialysis, in subsequent years, kidney transplantation was associated with substantially lower hospital care costs compared to maintenance dialysis.

In contrast to previous studies which reported increasing trends in healthcare costs by CKD stage [11,12], there was no evidence for a difference in annual hospital care costs between CKD stages 1-3B (mean eGFR = 37.6 mL/min/1.73 m^2^) and CKD stage 4 in SHARP. Patients with CKD stage 5 not on RRT were estimated to incur a small additional cost relative to earlier CKD stages, mainly attributable to access procedures performed in preparation for dialysis. There are a number of analytical differences between the SHARP cost study and previous studies that might explain this. First, unlike previous studies in which resource use and costs were summarized according to each patient’s baseline CKD stage, irrespective of further CKD progression, in SHARP costs were attributed to the actual stage of CKD at the end of each annual period. Second, in SHARP, the hospital costs of cardiovascular complications and deaths were evaluated separately from other hospital care. Third, this SHARP cost analysis focused on hospital care costs (including routine dialysis costs), but unlike in previous studies, other healthcare costs relevant to a CKD population (and increasing with kidney disease severity) such as costs for routine outpatient appointments, transport to and from hemodialysis units, primary care and prescription drugs [3,12-14] were not included since such information was not collected in SHARP or, in the case of prescription drugs, the collected information was not sufficient for costing.

SHARP recruited patients aged 40 years and older with moderate-to-severe CKD (mostly at stages 3B or worse) without a prior myocardial infarction or coronary revascularization. Consequently, SHARP is not representative of the full CKD population where CKD stage 1-3A predominates and the prevalence of coronary (and other comorbidities) is greater and increases with kidney disease severity [27]. As a consequence, SHARP patients in different CKD stages are likely to be more similar than is the case in the general CKD population. However, the SHARP estimation framework controls for vascular disease and diabetes at entry into the study as well as for CKD progression and cardiovascular disease occurring during follow-up, and, therefore, the hospital cost estimates corresponding to CKD stage and cardiovascular disease, are likely generalizable to a wider range of moderate-to-severe CKD adult patients, including those with pre-existing coronary disease.

A further potential limitation is that data on hospital resource use from the UK and other countries was used to inform UK costs. Preliminary work found no heterogeneity in hospital episode rates between countries conditional on events, except for patients recruited in Asia, who were excluded. The cost model using only UK patients produced similar results but was of limited power (1,935 patients were recruited in the UK). The UK hospital care costs in CKD, presented here, are likely also to be of limited generalizability to other countries contributing patient data for the analysis, as the respective costs of hospital services in these countries might differ from those in the UK.

## Conclusions

The UK annual hospital care costs model in moderate-to-severe CKD presented here identifies renal replacement therapy, major vascular events and deaths (vascular and non-vascular) as the key determinants of costs. The model allows reliable evaluation of effects of interventions and policies to modify rates of vascular events, progression of CKD, kidney transplant survival or access to transplantation in this population on hospital costs. To assist such efforts, a downloadable cost calculator is available at http://www.herc.ox.ac.uk/downloads/supportingmaterial.

## Additional file

Additional file 1: **Supplementary material to “What is the impact of chronic kidney disease stage and cardiovascular disease on the annual cost of hospital care kidney in moderate-to-severe disease?”.**

## Abbreviations

CKD: Chronic kidney disease
SHARP: Study of Heart and Renal Protection
UK: United Kingdom
RRT: Renal replacement therapy
MVE: Major vascular event
eGFR: Estimated glomerular filtration rate
FCE: Finished consultant episode
