# Supplementary material for: What is the impact of chronic kidney disease stage and cardiovascular disease on the annual cost of hospital care in moderate-to-severe kidney disease?
Source: BMC Nephrol. 2015 Apr 29;16:65. doi: 10.1186/s12882-015-0054-0 (PMC4424521; doi:10.1186/s12882-015-0054-0)
Supplement: Supplementary file 1 — Supplementary material to “What is the impact of chronic kidney disease stage and cardiovascular disease on the annual cost of hospital care kidney in moderate-to-severe disease?”. [file 12882_2015_54_MOESM1_ESM.docx]

**What is the impact of chronic kidney disease stage and cardiovascular disease on the annual cost of hospital care in moderate-to-severe kidney disease?**

SHARP Collaborative Group

**SUPPLEMENTARY MATERIAL**

| **Table S1. Serious adverse events related to hospital care use among the 7,246 SHARP participants contributing to the cost analysis** | |
| --- | --- |
| **Event category*** | **Event terms** |
| Any atherosclerotic | Above knee amputation; acute coronary syndrome/ hospitalisation with angina; acute ischaemic limb; amaurosis fugax/transient visual loss; amputation of finger/thumb; amputation of foot; amputation of toe; Aortic aneurysm; Aortic aneurysm dissection; aortic aneurysm repair or stent; Aortic aneurysm rupture; arterial embolism/thrombosis; arterial graft reconstruction/excision (not dialysis access); arterial surgery (not dialysis access); below knee amputation; carotid angioplasty stent; carotid surgery; CHD death (not MI); coronary angioplasty (PTCA) stent; coronary artery bypass graft (CABG); embolectomy; embolism/thrombosis arm artery; embolism/thrombosis leg artery; fem-pop bypass/leg artery bypass; heart failure - ischaemic; ischaemic/ gangrenous toe or finger; ischaemic/non-haemorrhagic stroke; ischaemic/non-haemorrhagic stroke - definite; Ischaemic/non-haemorrhagic stroke - presumed; leg artery angioplasty stent; limb ischaemia; myocardial infarction - definite; myocardial infarction - possible; myocardial infarction - probable; non coronary angioplasty stent; non coronary arterial surgery/intervention (not dialysis access); occluded arterial graft (not dialysis access); operation on/infection in/problem with amputation stump; popliteal or femoral or iliac aneurysm repair; post stroke complications; renal artery angioplasty stent; retinal artery occlusion/retinal thrombosis; stroke; transient ischaemic attack (TIA) (neurological symptoms < 24 hours); transplant renal artery angioplasty stent |
| Any non-atherosclerotic vascular | Angiogram of leg/femoral angiogram; aortic valve repair/replacement; arrhythmia; atrial fibrillation/flutter; bradycardia; cardiac arrest; cardiac congestion of liver; cardiomyopathy; cardiovascular investigations; cardioversion; carotid angiogram or arch aortagram; cerebral artery aneurysm surgery or clipping; conduction disorder/heart block; conduction system ablation; cor pulmonale or right heart failure; coronary angiogram/cardiac catheterisation; deep vein thrombosis (DVT); electrophysiological studies (EPS); giant cell arteritis/temporal arteritis; haemorrhagic stroke; heart failure - not ischaemic; heart failure/pulmonary oedema/congestive cardiac failure; heart valve problem; heart valve surgery; hypotension; internal cardiac defibrillator insertion/problem/battery change or check; ligation/stripping/injection of varicose veins; microscopic polyarteritis; mitral valve repair/replacement; non coronary angiogram; other cardiac death (not CHD); other cardiovascular procedures; pacemaker insertion/change/battery change; palpitations/fluttering of heart; pericardial effusion; pericardial surgery/pericardial drainage; pericarditis including Dressler's; postural hypotension; pulmonary embolism (PE) DVT; renal artery angiogram; spontaneous subdural haematoma; subarachnoid haemorrhage; sudden cardiac death; supraventricular tachycardia (SVT); tachycardia; temporal artery biopsy; uncontrolled hypertension/ stabilisation of blood pressure; vasculitis; venogram; venogram central; ventricular tachycardia (VT); Wegener's granulomatosis |
| Renal | Acute immunosuppressive medication toxicity; acute on chronic renal failure; acute on chronic renal failure not requiring dialysis; acute on chronic renal failure requiring dialysis; acute renal transplant dysfunction; acute renal transplant rejection; bladder biopsy/cystoscopy with biopsy; bladder investigations; bladder problem (except cancer); bladder surgery; bleeding from arteriovenous fistula/graft; blocked peritoneal dialysis catheter; blocked/thrombosed arteriovenous fistula/graft; cadaveric renal transplantation; calciphylaxis; change of dialysis modality; chronic allograft nephropathy; chronic immunosuppressive medication toxicity; chronic renal transplant rejection; chronic renal transplant dysfunction; conservative care for ESRD; creation of permanent arteriovenous fistula; creation of synthetic graft for dialysis; cystectomy; cystoscopy or urethroscopy; failed haemodialysis because of vascular access problem; fistulogram; fluid overload; haematuria; hyperkalaemia; hypokalaemia; hypotension on dialysis; ileal conduit surgery; infected arteriovenous fistula/graft; initiation of dialysis; initiation of haemodialysis; initiation of peritoneal dialysis; insertion of peritoneal dialysis catheter; insertion of temporary venous line; insertion of tunnelled venous line; insertion or removal trans-urethral or supra-pubic catheter; kidney investigations/procedure; kidney stone/ureter stone/bladder stone; living donor renal transplantation; localised dialysis catheter infection/abscess; localised peritoneal dialysis catheter infection/abscess; native kidney or renal transplant biopsy; native nephrectomy; nephrectomy; nephrostomystent/removal of renal stent; nephrotic syndrome; percutaneous angioplasty/embolectomy of arteriovenous fistula/graft; percutaneous or open kidney stone surgery/lithotripsy; peritoneal dialysis associated peritonitis; peritoneal dialysis catheter procedure; polycystic kidney disease; post renal transplant urinary sepsis; recurrence of primary renal disease in renal transplant; removal arteriovenous fistula/graft; removal of haemodialysis catheter; removal of peritoneal dialysis catheter; renal anaemia; renal colic or kidney pain; renal transplant complication; renal transplantation; sclerosing peritonitis; sepsis secondary to dialysis catheter infection; simultaneous kidney/pancreas transplantation; surgical exploration/repair arteriovenous fistula/graft; transplant nephrectomy; transurethral resection of bladder tumour (TURBT); uraemia; ureteric obstruction/hydronephrosis; ureteric surgery; urethral stricture/dilatation/surgery; urinary retention; venoplasty of central venous stenosis; withdrawal of dialysis |
| *The remaining ‘Other’ event category is omitted here due to the large number of event terms. | |

| **Table S2: CKD stage of patients at the end of analysis period (%) by baseline CKD stage** | | | | | | |
| --- | --- | --- | --- | --- | --- | --- |
|  | | **CKD stage at end of the study period for the cost analysis^1^** | | | | |
|  |  | **CKD 1-3B** | **CKD 4** | **CKD 5 not on dialysis** | **On dialysis** | **On transplant** |
| **Baseline CKD stage^2^** | **CKD 1-3B^3^** | 850 (57%) | 516 (35%) | 43 (3%) | 71 (5%) | 14 (1%) |
|  | **CKD 4** | 163 (7%) | 1,085 (49%) | 359 (16%) | 481 (22%) | 140 (6%) |
|  | **CKD 5 not on dialysis** | 6 (1%) | 40 (4%) | 171 (17%) | 589 (58%) | 211 (21%) |
|  | **On dialysis** | - | - | - | 1,891 (76%) | 607 (24%) |
| CKD = chronic kidney disease  ^1^Includes years up to an including the year prior to non-fatal censoring or year of death  ^2^9 patients who received a transplant prior to randomization were excluded from the tabulation by baseline CKD stage  ^3^Predominantly CKD stage 3B (eGFR ≥30 to <45 ml/min/1.73m²). | | | | | | |

| **Table S3. Number of hospital episodes excluding routine dialysis sessions (rate per patient per year) by baseline CKD stage** | | | | | | |
| --- | --- | --- | --- | --- | --- | --- |
| **Baseline CKD stage^1^** | **Number of patients** | **Any atherosclerotic** | **Any non-atherosclerotic vascular** | **Renal** | **Other (non-vascular and non-renal)** | **All episodes** |
| CKD stage 1-3B^2^ | 1,494 | 147 (0.02) | 200 (0.03) | 387 (0.06) | 1,433 (0.24) | 2,167 (0.36) |
| CKD stage 4 | 2,228 | 365 (0.04) | 391 (0.04) | 1,932 (0.22) | 2,406 (0.28) | 5,094 (0.58) |
| CKD stage 5 not on dialysis | 1,017 | 254 (0.07) | 241 (0.06) | 2,255 (0.59) | 1,243 (0.32) | 3,993 (1.04) |
| On dialysis | 2,498 | 765 (0.08) | 747 (0.08) | 4,280 (0.47) | 4,100 (0.45) | 9,892 (1.09) |
| **All** | 7,246 | 1,531 (0.06) | 1,579 (0.06) | 8,859 (0.32) | 9,188 (0.33) | 21,157 (0.77) |
| CKD = chronic kidney disease  ^1^9 patients who received a transplant prior to randomization were excluded from the tabulation by baseline CKD stage  ^2^Predominantly CKD stage 3B (eGFR ≥30 to <45 ml/min/1.73m²). | | | | | | |

| **Table S4. Predicted costs (95% confidence interval) [£] by current chronic kidney disease stage and cardiovascular disease history** | | | | | | | |
| --- | --- | --- | --- | --- | --- | --- | --- |
|  | **CKD 1-3B** | **CKD 4** | **CKD 5 not on dialysis** | **Kidney transplantation in the current annual period** | **Functioning kidney transplant from an earlier annual period** | **Maintenance dialysis initiated in the current annual period** | **Maintenance dialysis initiated in an earlier annual period** |
| **No death or vascular event during current annual period** | £403  (345-462) | £393  (343-444) | £525  (449-602) | £24,602  (24,027-25,178) | £1,148  (978-1,318) | £18,986  (18,620-19,352) | £23,326  (23,231-23,421) |
| **Vascular death in the current annual period** | £1,540  (871-2,209) | £1,530  (865-2,195) | £1,662  (995-2,328) | £25,739  (24,847-26,631) | £2,285  (1,599-2,970) | £11,320  (10,496-12,145) | £15,660  (14,915-16,405) |
| **Non-vascular death in the current annual period** | £1,795  (1,420-2,169) | £1,785  (1,413-2,156) | £1,917  (1,540-2,293) | £25,994  (25,303-26,684) | £2,539  (2,135-2,944) | £11,575  (10,866-12,285) | £15,915  (15,303-16,527) |
| **Non-fatal MVE during the current annual period** | £4,753  (4,226-5,280) | £4,743  (4,219-5,266) | £4,875  (4,347-5,402) | £28,952  (28,176-29,728) | £5,498  (4,931-6,064) | £25,119  (24,494-25,744) | £29,459  (28,941-29,976) |
| **Non-fatal MVE in the preceding annual period** | £1,142  (754-1,530) | £1,132  (744-1,519) | £1,264  (873-1,654) | £25,341  (24,637-26,044) | £1,886  (1,469-2,303) | £19,724  (19,197-20,252) | £24,064  (23,675-24,453) |
| **Non-fatal MVE two or more years previously or baseline vascular disease** | £575  (457-694) | £565  (448, £683) | £697  (570-824) | £24,774  (24,184-25,365) | £1,320  (1,120-1,520) | £19,158  (18,780-19,536) | £23,498  (23,365-23,630) |
| UK 2011 costs are presented for an individual without diabetes; diabetes is estimated to increase all the above costs by £171. CKD = chronic kidney disease | | | | | | | |

**Statistical appendix**

*Model selection*

The annual hospital cost data in SHARP possesses features typical of cost data: a large proportion of zero cost observations (42%), and the positive cost observations possessing a multimodal and right-skewed distribution. A range of two-part and single-equation models were considered for modelling annual hospital costs. Generalised linear models were used in the second part of the two-part models and in the single-equation models. Recommended procedures for identifying appropriate variance and link functions in generalised linear models were adopted [[1-4](#_ENREF_1)]. Five candidate single equation and two-part models that most closely satisfied the appropriate variance and link functions, or are commonly used in applied work, were compared in terms of their predictive performance using the following metrics of fit [[2](#_ENREF_2)]: mean prediction error (MPE), mean absolute prediction error (MAPE), and root mean square error (RMSE). These metrics of model fit were calculated using a cross-validation process in which the individual patients’ data was randomly split 1,000 times into estimation and validation samples (2/3 individuals in estimation sample and 1/3 in the validation sample); all models were estimated on the estimation sample and the metrics calculated on the validation sample, and then averaged across the 1,000 random samples. The metrics of fit were assessed in the overall sample as well as in quintiles of study participants defined by estimated 5-year major vascular event (MVE) risk [[5](#_ENREF_5)].

The five models considered were: (1) Single-equation model with an identity link and a Gaussian variance; (2) Two-part model with a second part model with identity link and a Gaussian variance; (3) Single-equation model with an identity link and a poisson variance; and (4) Two-part model with a second part model with a square root link and a poisson variance, and; (5) Two-part model with a second part model specified with a log link and gamma variance (the most commonly used model in recent applied costing literature).

Table S5 presents the metrics of fit from the cross-validation process for the 5 candidate models at the overall sample level. Except for the two-part log-gamma GLM, which performed comparatively poorly on all metrics of fit, the remaining models performed similarly on all criteria. The assessment of model fit in quintiles of CVD risk, also did not identify a clearly preferred statistical model, with the single-equation and two-part models performing better in different parts of the distribution in terms of MPE, and similarly across the remaining measures of fit. The single equation linear regression model with an identity link and a Gaussian variance was chosen based on the principle of parsimony – i.e. the simplest best model.

*Covariate selection*

Initially, a full model was specified including the full hierarchical cardiovascular complication categorisation and the categorisation of chronic kidney disease stage (see main text), and a large number of individual clinical measurements and demographic characteristics (see Table S6). Backward-stepwise selection using likelihood ratio tests was applied to these demographic characteristics and clinical measurements using a cut-off p-value of 0.01 to account for the multiple testing and large sample size. After adjusting for cardiovascular complications and chronic kidney disease stage, the single further characteristic retained in the linear regression model was the individual’s prior history of diabetes. As a sensitivity analysis, selection of covariates was repeated 1,000 times using a bootstrap technique, and the proportion of times each covariate was retained in the model was recorded.

Alternative specifications were considered for the annually-updated cardiovascular complications and stage of chronic kidney disease. There was no evidence for a differential effect across the following categories: MVE two years previously; more than two years previously; and history of vascular disease at entry into the study (p-value = 0.13 based on an F-test of coefficient equality). Hence, these were combined to form a new cardiovascular disease category.

Interactions between separately being ‘on dialysis’ or ‘on a functional kidney transplant’ and death or experiencing non-fatal MVEs in the current year were considered and retained if with a p-value of less than 0.001. Two interactions were retained in the final model: (1) being on dialysis and experiencing a fatal-event and (2) being on dialysis and experiencing a non-fatal MVE in the current period.

| **Table S5. Overall metrics of fit from cross-validation procedure** | | | |
| --- | --- | --- | --- |
|  | **MPE** | **MAPE** | **RMSE** |
| Single-equation: normal variance, identity link | -1 (0.0) | 2,022 (20.3) | 3,669 (36.8) |
| Two-part: normal variance, identity link | -1 (0.0) | 2,022 (20.3) | 3,669 (36.8) |
| Single-equation: poisson variance, identity link | -1 (0.0) | 2,017 (20.2) | 3,669 (36.8) |
| Two-part: poisson variance, square-root link | -1 (0.0) | 2,021 (20.3) | 3,671 (36.8) |
| Two-part: gamma variance, log link | -18 (-0.2) | 2,042 (20.5) | 3,768 (37.8) |
| Numbers are metric of fit (percentage of mean cost). All metrics are in £.  MPE=mean prediction error; MAPE=mean absolute prediction error; RMSE=root mean squared error | | | |

| **Table S6: Baseline covariates considered in the automatic selection procedure for the cost model** | | |
| --- | --- | --- |
| **Variable** | **Type** | **Categories/Description** |
| Age (annually-updated) | Continuous | age at start of annual period |
| Gender | Binary | male; female |
| Treatment allocation | Binary | ezetimibe/simvastatin; placebo |
| Smoking status | Categorical | never; former; current |
| Diabetes | Binary | yes; no |
| Failed transplant at randomisation | Binary | yes; no |
| Current alcohol drinker | Binary | yes; no |
| Adult dependants | Binary | yes; no; missing |
| Child dependants | Binary | yes; no; missing |
| Education level | Categorical | A-levels and above; GCSE/vocational; below secondary; missing |
| Renal diagnosis at baseline | Categorical | diabetic nephropathy; cystic kidney disease; other (known or unknown cause) |
| Body mass index | Categorical | < 24.5 kg/m^2^; 24.5-28.4 kg/m^2^; > 28.4 kg/m^2^; missing |
| Diastolic blood pressure | Categorical | < 73 mmHg; 73-84 mmHg; > 84 mmHg; missing |
| Systolic blood pressure | Categorical | < 129 mmHg; 129-147 mmHg; > 147 mmHg; missing |
| Albumin level | Categorical | < 3.8 g/dL; 3.8-4.2 g/dL; > 4.2 g/dL; missing |
| Urinary albumin:creatinine ratio | Categorical | < 30 mg/g; 30-300 mg/g; > 300 mg/g; missing |
| Haemoglobin | Categorical | < 11.6 g/dL; 11.6-13.0 Hg; > 13.0 Hg; missing |
| Phosphate | Categorical | 1.2-1.5 mmol/L; > 1.5 mmol/L; missing; missing |
| Total cholesterol | Categorical | < 4.3 mmol/L; 4.3-5.3 mmol/L; > 5.3 mmol/L; missing |
| LDL cholesterol | Categorical | < 2.4 mmol/L; 2.4-3.1 mmol/L; > 3.1 mmol/L; missing |
| HDL cholesterol | Categorical | < 0.9 mmol/L; 0.9-1.2 mmol/L; > 1.2 mmol/L; missing |
| Continuous variables (excluding age) were split into three categories by the approximate tertiles of the distributions. For all variables, where the percentage of missing data was low (<5%), missing observations were allocated to the middle tertile; otherwise a separate missing category was included. | | |

**Statistical appendix references**

1. Manning WG, Mullahy J. Estimating log models: to transform or not to transform? Journal of health economics 2001;20(4):461-494

2. Jones A. Health Econometrics. In: Culyer AJ, Newhouse JP, eds. *Handbook of Health Economics*. Elsevier: Amsterdam; 2000.

3. Hosmer DW, Lemeshow S. Goodness of Fit Tests for the Multiple Logistic Regression Model. Communications in Statistics –Theory and Methods 1980;9(10):1043-1069

4. Pregibon D. Goodness of link tests for generalized linear models. Applied Statistics 1980;29:15-24

5. Mihaylova B, Emberson J, Blackwell L*, et al.* The effects of lowering LDL cholesterol with statin therapy in people at low risk of vascular disease: meta-analysis of individual data from 27 randomised trials. Lancet 2012;380(9841):581-590
